# Analysis of ferritinophagy-related genes associated with the prognosis and regulatory mechanisms in non-small cell lung cancer

**DOI:** 10.3389/fmed.2025.1480169

**Published:** 2025-03-07

**Authors:** Yuan Hao, Xin Wang, Zerong Ni, Yuhui Ma, Jing Wang, Wen Su

**Affiliations:** ^1^Clinical Trials Center, Cancer Hospital Affiliated to Shanxi Medical University, Shanxi Province Cancer Hospital, Shanxi Hospital Affiliated to Cancer Hospital, Chinese Academy of Medical Sciences, Taiyuan, China; ^2^Department of Cancer Center, Shanxi Bethune Hospital, Shanxi Academy of Medical Sciences, Tongji Shanxi Hospital, Tongji Medical College Huazhong University Science of and Technology, Taiyuan, China; ^3^Department of Pathology, Shanxi Cancer Hospital, Shanxi Hospital Affiliated to Cancer Hospital, Chinese Academy of Medical Sciences, Cancer Hospital Affiliated to Shanxi Medical University, Taiyuan, China; ^4^Department of Immunology, Cancer Hospital Affiliated to Shanxi Medical University, Shanxi Province Cancer Hospital, Shanxi Hospital Affiliated to Cancer Hospital, Chinese Academy of Medical Sciences, Taiyuan, China

**Keywords:** non-small cell lung cancer, ferritinophagy, TCGA, machine learning, prognosis

## Abstract

Lung cancer remains a major global health issue, with non-small cell lung cancer (NSCLC) constituting approximately 85% of cases. Ferritinophagy, a pivotal autophagic process in ferroptosis, plays an essential role in tumor initiation and progression. However, the specific contributions of ferritinophagy-related genes (FRGs) to NSCLC pathogenesis remain incompletely understood. In this study, weighted gene co-expression network analysis (WGCNA) was employed to identify key modular genes associated with FRG scores. Genes overlapping between these modules and differentially expressed genes (DEGs) were selected for further investigation. Prognostic genes were identified through univariate Cox regression and least absolute shrinkage and selection operator (LASSO) analysis, with subsequent validation using quantitative reverse transcriptase polymerase chain reaction (qRT-PCR) on both clinical samples and the TCGA-NSCLC dataset. A nomogram incorporating clinicopathological features and risk scores was developed to predict patient outcomes. Further analyses focused on functional enrichment, drug sensitivity, and the immune microenvironment. Cross-referencing 2,142 key modular genes with 2,764 DEGs revealed 600 candidate genes. Univariate Cox regression and LASSO analysis of these candidates identified eight prognostic genes: KLK8, MFI2, B3GNT3, MYRF, CREG2, GLB1L3, AHNAK2, and NLRP10. Two distinct risk groups exhibited significant survival differences. Both the risk score and pathological N stage were found to be independent prognostic factors, forming the basis for the nomogram. Notable correlations were observed between certain immune cells, prognostic genes, and immune responses, affecting the efficacy of immunotherapy and drug sensitivity. qRT-PCR confirmed that, except for NLRP10, all prognostic genes exhibited expression patterns consistent with TCGA-NSCLC data. This study highlights the significant role of FRGs in NSCLC prognosis and regulation, offering novel insights for personalized treatment strategies.

## 1 Introduction

Lung cancer remains a significant global health threat, with non-small cell lung cancer (NSCLC) accounting for roughly 85% of all cases (1). Recent advancements in molecular targeted therapies have brought new hope for NSCLC treatment. Drugs such as osimertinib, which targets EGFR mutations, lorlatinib for ALK rearrangements, dabrafenib for BRAF V600E mutations, and adagrasib for KRAS G12C mutations, have demonstrated clinical efficacy. Despite these developments, the 5 years survival rate for NSCLC remains below 18% (2). Concurrently, PD-1/PD-L1 inhibitors have been approved for the first-line treatment of advanced NSCLC; however, their response rate is only around 20% in unselected patients with advanced disease (3–5). These challenges underscore the ongoing need to identify novel mechanisms and therapeutic targets to improve prognosis and treatment outcomes for NSCLC.

In recent years, ferroptosis has garnered significant attention for its pivotal role in the progression of malignant tumors (6). First described by Stockwell, ferroptosis is a regulated form of cell death that diverges from apoptosis, necrosis, and autophagy in terms of its morphological, biochemical, and genetic characteristics (7). This process is primarily characterized by disruptions in iron homeostasis, alongside the accumulation of reactive oxygen species (ROS) and lipid peroxides, which ultimately lead to cell death (8, 9) Central to the regulation of ferroptosis is ferritinophagy, a selective autophagy process that controls intracellular iron homeostasis. This process is particularly significant in cancer cells, as it directly influences the availability of free iron, a key driver of ferroptosis (10–13). In the context of NSCLC, understanding ferritinophagy is crucial as it may provide new insights into tumor progression and resistance mechanisms, while also offering potential targets for therapeutic intervention.

Recent studies have highlighted the significant impact of ferritinophagy on the development and progression of various cancers. For instance, Zhao et al. (14) demonstrated that NCOA4-mediated ferritinophagy activates the JNK pathway, inducing ferroptosis in colorectal cancer cells. Similarly, Santana-Codina et al. (15) revealed that ferritinophagy, which is upregulated in pancreatic cancer, supports tumor growth by maintaining iron levels and enhancing mitochondrial function. Elevated ferritinophagy activity correlates with accelerated tumor growth and poorer outcomes. In liver cancer, Xiu et al. (16) showed that inducing NCOA4-mediated ferritinophagy significantly inhibits tumor progression in both *in vivo* and *in vitro* models. Emerging evidence also suggests that ferritnophagy plays a pivotal role in NSCLC progression and drug resistance. For instance, NCOA4, a key mediator of ferritinophagy, is upregulated in osimertinib-resistant NSCLC cells, promoting ferritinophagy and contributing to adaptive resistance (17). Similarly, DTX2, a ubiquitin ligase, negatively regulates NCOA4-mediated ferritinophagy, and its knockdown enhances cisplatin-induced ferroptosis and overcomes drug resistance in NSCLC (18). COPZ1 silencing has also been shown to promote NCOA4-mediated ferritinophagy, triggering ferroptosis in lung adenocarcinoma cells (19). Additionally, interferon (IFN)-γ-induced TFR1 upregulation promotes ferritinophagy and ferroptosis in NSCLC, suggesting a potential synergy between immune signaling and iron metabolism therapies (20). Furthermore, the compound ShtIX induces ferroptosis in NSCLC cells through NCOA4-mediated ferritinophagy, highlighting the therapeutic potential of targeting this pathway (21).

These findings collectively emphasize the significance of ferritinophagy in regulating ferroptosis and its potential as a therapeutic target in NSCLC. This study aims to further explore the prognostic and regulatory roles of ferritinophagy-related genes in NSCLC, providing new insights into the molecular mechanisms underlying ferroptosis and its therapeutic implications.

## 2 Materials and methods

### 2.1 Data source

The datasets for TCGA-Lung Adenocarcinoma (LUAD) and Lung Squamous Carcinoma (LUSC) were initially sourced from the UCSC Xena website^1^ and subsequently merged into The Cancer Genome Atlas Non-Small Cell Lung Cancer (TCGA-NSCLC) dataset. This dataset included 1,025 primary tumor samples (NSCLC) and 108 paraneoplastic normal tissue samples (Normal), comprising gene expression data for 1,004 individuals with available overall survival (OS) data (Dead: 396, Alive: 608), as well as associated clinical data. Additionally, the GSE37745 dataset (GPL570), containing 196 NSCLC tissue samples, was retrieved from the Gene Expression Omnibus (GEO) database^2^. Furthermore, 20 ferritinophagy-related genes (FRGs) were identified using the GeneCards database^3^ (22).

### 2.2 Single sample gene set enrichment analysis (ssGSEA) and weighted gene co-expression network analysis (WGCNA)

In order to explore the relationship between FRGs and the prognosis of NSCLC patients, based on 20 FRGs, the ssGSEA method of GSVA (v 1.42.0) was used to generate FRGs-related ssGSEA enrichment scores for 1004 NSCLC samples with survival data in the TCGA-NSCLC dataset (23). The samples were categorized into high and low scoring groups based on optimal cut-off values for the scores using the survminer::surv_cutpoint function with minprop = 0.1. Kaplan-Meier (KM) survival analysis was conducted between these groups using the R package survminer (v 0.4.9) (*p* < 0.05) (24).

Subsequently, to explore the relationship between co-expressed gene modules and FRGs, the WGCNA (v 1.71) (25) was applied to the TCGA-NSCLC dataset to identify genes associated with FRG scores in NSCLC. Hierarchical clustering, based on the Euclidean distance of expression, was first performed to identify and exclude outliers. A scale-free network was constructed with a minimum of 500 genes per module, applying a soft threshold where R2 exceeded 0.85 and mean connectivity approached zero. Then, the ssGSEA scores related to FRGs were used as the phenotype to construct a phenotype-module correlation heatmap, and todules exhibiting the highest positive and negative correlations with the scores were selected as key modules, and genes within these modules were identified for further analysis.

### 2.3 Differential expression and functional enrichment analysis

Differentially expressed genes (DEGs) between tumor and normal tissue groups were identified using DESeq2 (v 1.34.0) (26) with the following criteria: p.adj < 0.05 and | log_2_Fold Change (FC)| > 2.0. In order to understand the distribution of differentially expressed genes as a whole, the DEGs were visualized using the R packages ggplot2 (v 3.4.1) (27) and ComplexHeatmap (v 2.15.1) (28).

Overlapping genes between the differentially expressed genes (DEGs) and key module genes were identified as candidate genes, and Venn diagrams were generated using the VennDiagram package (v 1.7.1) (29). Gene Ontology (GO) and Kyoto Encyclopedia of Genes and Genomes (KEGG) enrichment analyses were performed on these candidate genes with the clusterProfiler R package (v 4.2.2) (30) to identify functional pathways (p.adj < 0.05). Additionally, a medium confidence score of 0.4 was applied in the STRING database^4^ to explore potential protein-protein interactions (PPIs) among the proteins encoded by the candidate genes and construct a PPI network.

### 2.4 Construction and validation of risk models

To evaluate whether prognostic genes were associated with patient survival, the expression levels of candidate genes were used as continuous variables to correlate with survival information for each sample, with survival outcomes and survival time as the dependent variables. Univariate Cox analysis (hazard ratios (HR) ≠ 1 and *p* < 0.05) was performed on the candidate genes using the survival (v 3.3-1) and survminer R packages. A proportional hazards (PH) hypothesis test was subsequently applied (*p* > 0.05). Genes passing the PH hypothesis test were designated as candidate prognostic genes. LASSO (Least Absolute Shrinkage and Selection Operator) analysis was then applied to these candidate genes using the glmnet R package (v 4.1-2) (31) to identify the genes that best fit the model with the smallest error. During the model training process, 10-fold cross-validation (CV) was used to obtain the optimal Lambda (lambda.min), corresponding to the value that produced the smallest cross-validation error. Genes with regression coefficients that were not penalized to zero were selected as prognostic genes and risk scores were computed using the corresponding formula:


$$\text{RiskScore}=\sum_{i=1}^{n}{\text{Coef}}_{\text{i}}\,\text{×}\,{\text{X}}_{\text{i}}$$

The coefficients (Coef) and gene expression values (X) representing the model parameters.

Risk scores for NSCLC samples from both the TCGA-NSCLC and GSE37745 datasets were calculated, and the samples were divided into high- and low-risk groups based on optimal cutoff values. KM survival analysis was performed to compare survival differences between these groups using the survminer package. In parallel, risk score distributions and risk curves were visualized. To assess the prognostic performance, survival ROC analysis (v 1.0 3.1) (32) was performed to generate receiver operating characteristic (ROC) curves, with areas under the curve (AUC) exceeding 0.6 for predicting 1, 3, and 5 years survival outcomes in patients with NSCLC.

### 2.5 Independent prognostic analysis

To determine independent prognostic factors for NSCLC, univariate Cox regression analysis (*p* < 0.05) was conducted combining risk scores with clinical features, including age, gender, pathologic T, pathologic N, and pathologic M stages. These variables were tested for the PH hypothesis, and factors passing the test were subjected to multivariate Cox regression analysis to identify independent prognostic factors (*p* < 0.05). Based on these factors, a nomogram was constructed using the rms package (v 6.2-0) to predict 1, 2, and 3 years survival probabilities in patients with NSCLC. The predictive accuracy of the nomogram was validated using calibration curves and ROC curves.

### 2.6 Functional enrichment analysis

To explore the functional pathways associated with prognostic genes, GSEA was performed separately for the two risk groups and individual prognostic genes. The log_2_FC between the two groups were calculated using DESeq2, with differential genes ranked by log_2_FC from highest to lowest. The cc2.cp.kegg.v7.4.symbols.gmt file from the Molecular Signatures Database (MSigDB^5^) served as the background gene set, and GSEA was conducted with clusterProfiler (*p* < 0.05). Additionally, Spearman correlations were calculated between prognostic genes and other genes in TCGA-NSCLC, with correlation coefficients ranked from highest to lowest, followed by GSEA (*p* < 0.05).

### 2.7 Immune microenvironmental analysis in NSCLC

To assess the involvement of immune cells in NSCLC, immune cell infiltration was analyzed using the immunedeconv package (v 2.0.4) (33), excluding samples with *p* > 0.05. Differences between the two risk groups were compared, and Spearman’s correlation was calculated between prognostic genes and differential immune cells (*p* < 0.05). Data on tumor-associated major histocompatibility complex molecules, chemokines, immunosuppressive, and immunostimulatory factors were retrieved from the TISIDB database^6^ and analyzed for correlations with prognostic genes and risk scores using Spearman’s correlation.

The ssGSEA algorithm in the GSVA package (v 1.42.0) (23) was employed to compute immunotherapy pathway scores for NSCLC samples and examine the relationship between risk scores and immune pathways. Differences in immune checkpoint inhibitor (ICI) gene expression between risk groups were assessed using the Wilcoxon test (*p* < 0.05), with further evaluation of their correlation with risk scores. Immunophenoscores (IPS) from The Cancer Immunome Atlas (TCIA)^7^ were used to predict responses of the two risk groups to anti-CTLA-4 and anti-PD-1 therapies (*p* < 0.05).

### 2.8 Drug sensitivity analysis and prognostic gene expression verification

To evaluate differences in drug sensitivity, the half-maximal inhibitory concentration (IC50) of chemotherapeutic agents was assessed between the two risk groups using the pRRophetic algorithm (v 0.5) (34). Additionally, the expression of prognostic genes was compared between tumor and normal samples from both the TCGA-NSCLC dataset and clinically collected specimens.

### 2.9 Quantitative reverse transcriptase polymerase chain reaction (qRT-PCR)

The expression of prognostic genes was also analyzed in malignant tissues from five patients with NSCLC and matched normal tissues from five individuals at Shanxi Province Cancer Hospital. For RNA extraction, 50 mg of tissue from each sample was combined with 1 mL of TRIzol reagent (Ambion, United States). RNA concentration was measured, and reverse transcription was performed according to the manufacturer’s instructions. RNA concentration was determined by extracting 1 μL and using a NanoPhotometer N50. cDNA synthesis was performed using the SureScript First-Strand cDNA Synthesis Kit (Servicebio, China), and the resulting cDNA was diluted 5–20 times with ddH_2_O. qPCR was carried out for 40 cycles (initial denaturation at 95°C for 1 min, denaturation at 95°C for 20 s, annealing at 55°C for 20 s, and extension at 72°C for 30 s). Gene expression was normalized to GAPDH using the 2^–ΔΔCT^ method. Primer information is provided in Table 1.

**TABLE 1:** Sequence information of polymerase chain reaction (PCR) primers.

Primer	Sequences
KLK8 F	GCCGTGTGTCCATTTGAACC
KLK8 R	AGCTGTAAGGACCCAGTTGC
MFI2 F	CACAGCAGTGAGCGAGTTCT
MFI2 R	CAAAGACGGTTGTGTGCCTG
B3GNT3 F	AAACTCTTTCTTCGGCTCGC
B3GNT3 R	GGGAACGCCGGAGACAATTA
MYRF F	TGGACCTGCCATCAGTGTCT
MYRF R	TGGACCTGCCATCAGTGTCT
CREG2 F	TGATGCAGGCCCCTTTATCTG
CREG2 R	AAGGACGAGGGGATCTCACA
GLB1L3 F	AGTGCATCTCGATACCTCCCT
GLB1L3 R	ATGGGAGATGGAAAGGCGTC
AHNAK2 F	GCGTCTGTAGCTTCCTTGTG
AHNAK2 R	TCCGTGAGTCCCCTGAATCT
NLRP10 F	GTAGGTACCAGCACCACCAA
NLRP10 R	GTAAGCAAAGCCTGGGGACT
Endogenous-GAPDH F	CGAAGGTGGAGTCAACGGATTT
Endogenous-GAPDH R	ATGGGTGGAATCATATTGGAAC

### 2.10 Statistical analysis

Machine learning and bioinformatics analyses were performed using R statistical software (version 4.2.2). The Wilcoxon test (*p* < 0.05) was applied to compare data between groups.

## 3 Results

### 3.1 The 2,142 key modular genes related to FRG scores were discovered in NSCLC

**FIGURE 1:**
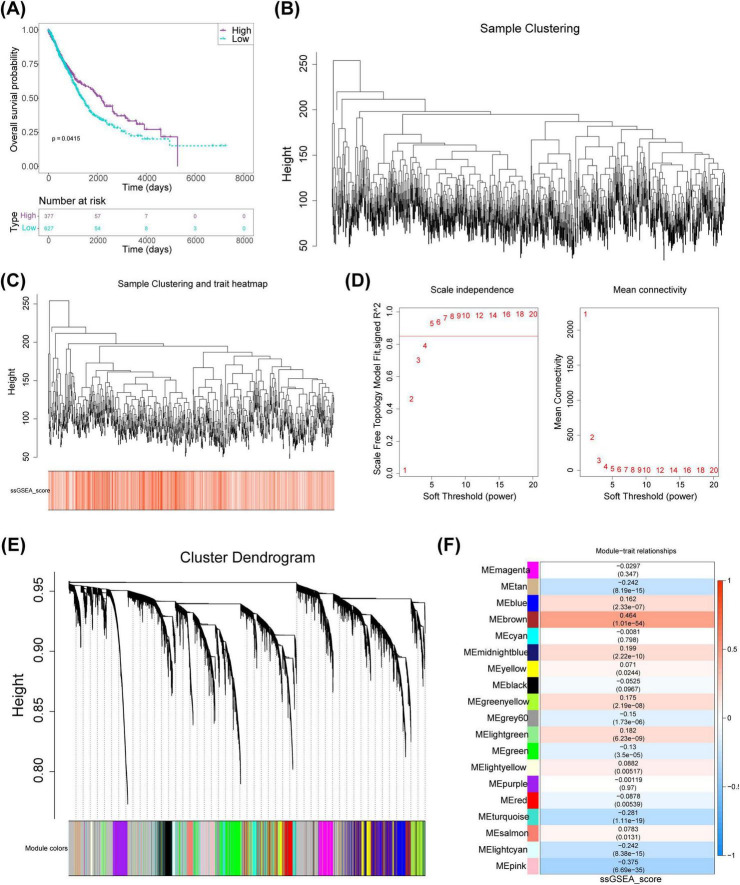
Identification of key modular genes associated with FRGs scores in NSCLC. **(A)** FRGs-related KM curves. **(B)** Clustering tree for NSCLC samples. **(C)** Hierarchical clustering of samples with integrated trait information. **(D)** Soft threshold selection for network analysis. **(E)** Co-expressed modular genes identified *via* WGCNA. **(F)** Heatmap depicting correlations between phenotype and module genes. KM, Kaplan-Meier; NSCLC, non-small cell lung cancer; WGCNA, weighted gene co-expression network analysis; FRGs, ferritinophagy-related genes.

Scores were calculated using FRGs in the TCGA-NSCLC dataset. Subsequently, NSCLC samples were categorized into high- and low-scoring groups (cutoff value = 2.148193). The analysis revealed a significant survival difference between the two groups, with the high-scoring cohort demonstrating a markedly higher survival rate than the low-scoring group, indicating a potential association between FRGs and NSCLC prognosis (Figure 1A). WGCNA was then employed to identify genes associated with FRG scores. After excluding an outlier sample (Figures 1B, C), soft threshold screening was conducted, as shown in Figures 1A, D threshold value of five was determined as optimal based on R^2^ and mean connectivity. Hierarchical clustering subsequently uncovered nine gene modules (Figure 1E), among which the pink module (r = -0.375, 900 genes) and brown module (r = 0.464, 1,242 genes) were strongly negatively and positively correlated with scores, respectively (Figure 1F). The genes from these two modules were merged, resulting in a set of 2,142 key module genes for further investigation.

### 3.2 The 600 candidate genes had a strong connection with cellular functional activity

**FIGURE 2:**
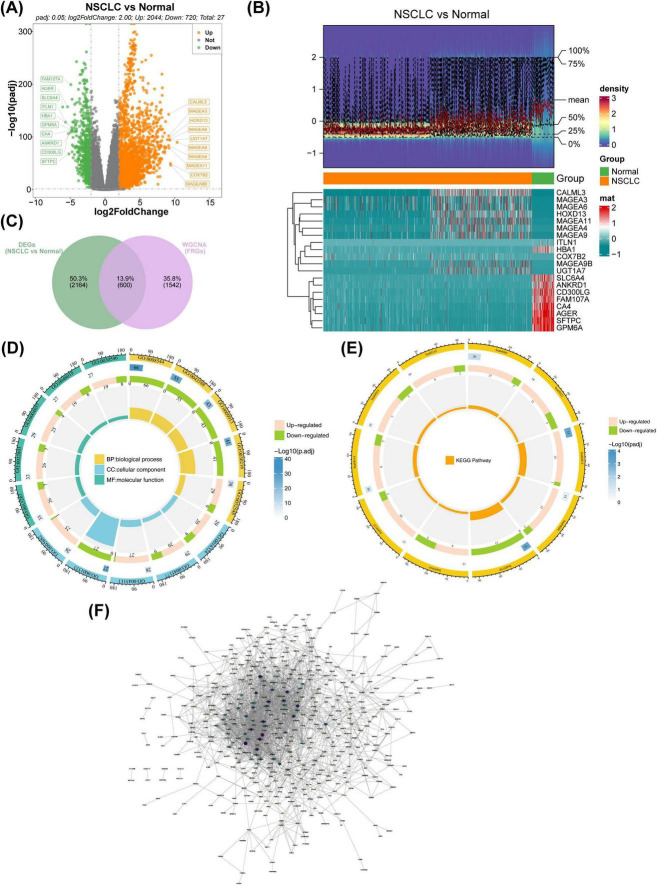
Identification of candidate FRGs. **(A)** Volcano plot illustrating the distribution of genes between NSCLC and normal samples. Red represents upregulated genes, blue denotes downregulated genes, and gray indicates non-significant genes. **(B)** Heatmap visualizing differential gene expression between NSCLC and normal groups. Red indicates high expression density, while blue indicates low expression density. **(C)** Venn diagram showing the overlap between differentially expressed genes (DEGs) and WGCNA-identified genes. **(D)** GO enrichment analysis of candidate genes depicted in a circular map. **(E)** KEGG enrichment analysis of candidate genes shown in a circular map. **(F)** PPI network analysis for candidate genes. FRGs, ferritinophagy-related genes; NSCLC, non-small cell lung cancer; GO, gene ontology; KEGG, Kyoto Encyclopedia of Genes and Genomes; WGCNA, weighted gene co-expression network analysis.

Differential expression analysis in TCGA-NSCLC identified 2,764 DEGs between NSCLC and normal samples, with 2,044 upregulated and 720 downregulated genes (Figures 2A, B). Upon intersecting the DEGs with the key modular genes, 600 candidate genes were identified (Figure 2C). Functional enrichment analysis revealed these candidate genes to be involved in 143 GO terms, including apical plasma membrane, epidermis development, and channel activity, as well as 217 pathways such as the PI3K-Akt signaling pathway and Neuroactive ligand-receptor interaction (Figures 2D, E). Furthermore, the PPI network indicated complex interactions among the proteins encoded by these candidate genes, including SPRR2E-SPRR2B and CBR3-DSC1, among others (Figure 2F).

### 3.3 A risk model created using prognostic genes reliably predicts survival in patients with NSCLC

**FIGURE 3:**
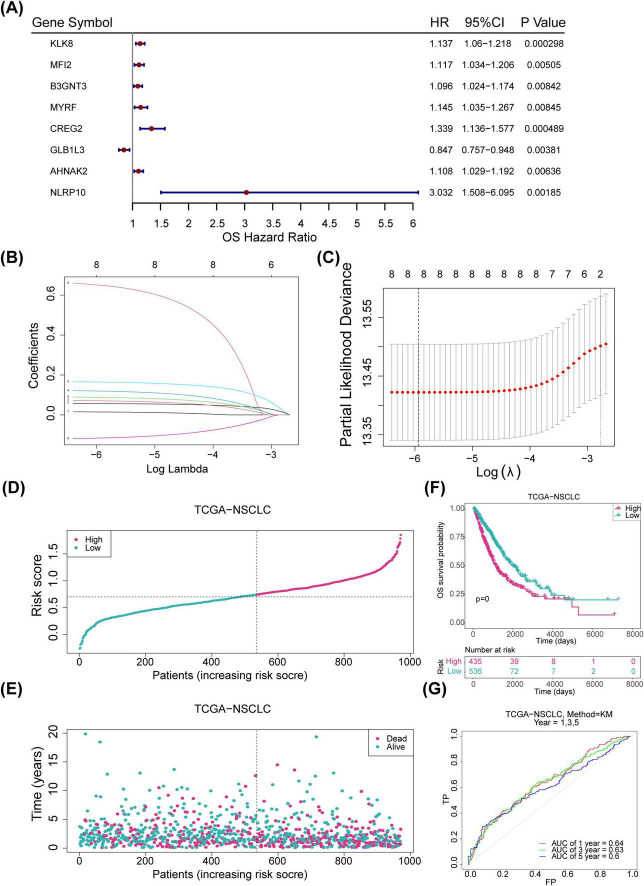
Construction of risk models. **(A)** Forest plot displaying one-factor Cox regression results for the internal training set (*p* < 0.05). **(B)** LASSO regression coefficients for eight genes. **(C)** LASSO model residual sum of squares for the eight genes. **(D)** Risk profiles for the training set, with red indicating high-risk samples and blue indicating low-risk samples. **(E)** Survival status plot for the training set, with red representing deceased samples and blue representing survivors. **(F)** Kaplan-Meier (KM) survival curves for the training set. **(G)** ROC curve for the training set. LASSO, least absolute shrinkage and selection operator; ROC, receiver operating characteristic.

**FIGURE 4:**
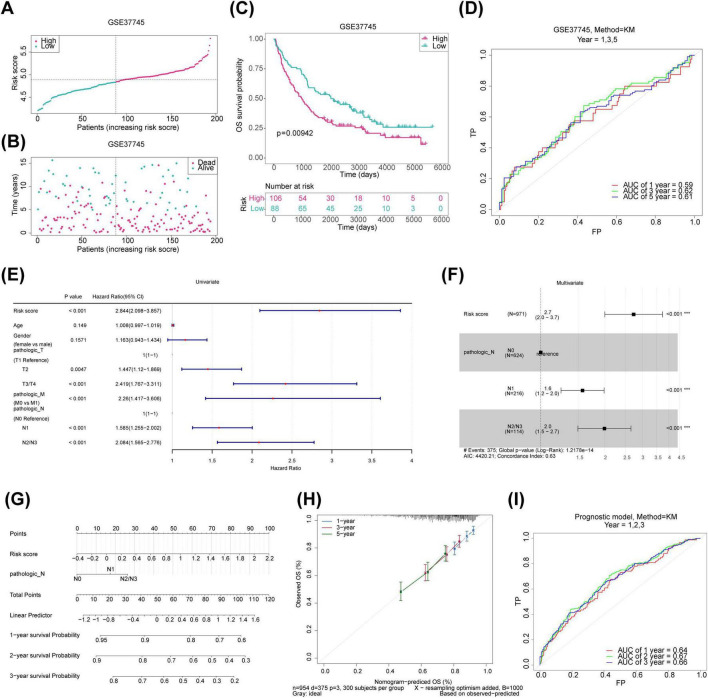
Validation of risk model and construction of prognostic prediction models for the training set. **(A)** Risk profile plot for the validation set, with red dots indicating high-risk samples and blue dots representing low-risk samples. **(B)** Survival status plot for the validation set, with red representing deceased samples and blue indicating survivors. **(C)** KM survival curves for the validation set. **(D)** ROC curve for the validation set. **(E)** Forest plot of univariate Cox regression for clinical indicators in the training set. **(F)** Forest plot of multivariate Cox regression for clinical indicators in the training set. **(G)** Nomogram column plot for prognostic prediction. **(H)** Calibration curves for evaluating prognostic models. **(I)** ROC curves for the prognostic prediction models. KM, Kaplan-Meier; ROC, receiver operating characteristic.

Univariate Cox regression and PH assumption tests of the 600 candidate genes identified eight genes (KLK8, MFI2, B3GNT3, MYRF, CREG2, GLB1L3, AHNAK2, and NLRP10) with *P* < 0.05 (Figure 3A). All 8 genes passed the PH assumption test (*P* > 0.05) and were selected as significant prognostic candidates (Figure 3A). LASSO analysis incorporating these genes revealed minimal model error (λmin = 0.00263837), confirming their role as prognostic markers (Figures 3B, C). Risk scores were calculated from the expression levels and coefficients of these genes using the TCGA-NSCLC data: *RiskScore*=*KLK*8×(0.0570633314496996)+*MFI*2×(0.0703586594018025)+*B*3*GNT*3×(0.0877327800833761)+*MYRF*×(0.119365343024516)+*CREG2*(0.164451250257509)+*GLB1L3*(−0.116152759959115)+*AHNAK2*(0.0144952367245304)+*NLRP10*(0.649738673795027). Optimal cutoff values for risk scores stratified samples into high- and low-risk groups in both the TCGA-NSCLC (Risk score = 0.284514) and GSE37745 datasets (Risk score = 4.83772). Higher risk scores were associated with increased NSCLC mortality, and survival curves corroborated poorer outcomes for the high-risk group (Figures 3D–F). The survival AUC values at 1, 3, and 5 years surpassed 0.6, indicating reasonable accuracy of the risk model in predicting patient survival (Figure 3G). Similar trends were observed in the GSE37745 dataset (Figures 4A–D).

### 3.4 A nomogram created based on independent prognostic factors accurately predicts the survival in NSCLC

To identify independent prognostic factors for NSCLC, a univariate Cox regression analysis was performed on six variables: gender, age, pathologic T, pathologic N, pathologic M, and risk score. Pathologic T, pathologic N, pathologic M, and risk score showed significant associations with prognosis (pathologic T and pathologic M were excluded after failing the PH assumption test) (Figure 4E). Pathologic N and risk score were then integrated into a multivariate Cox analysis, resulting in a prognosis model where pathologic N1, pathologic N2/N3, and risk score emerged as independent prognostic factors for NSCLC (Figure 4F). Calibration and ROC curves affirmed the nomogram’s accuracy in predicting survival based on these independent factors (Figures 4G–I).

### 3.5 Possible functional regulation among eight prognostic genes screened in NSCLC

**FIGURE 5:**
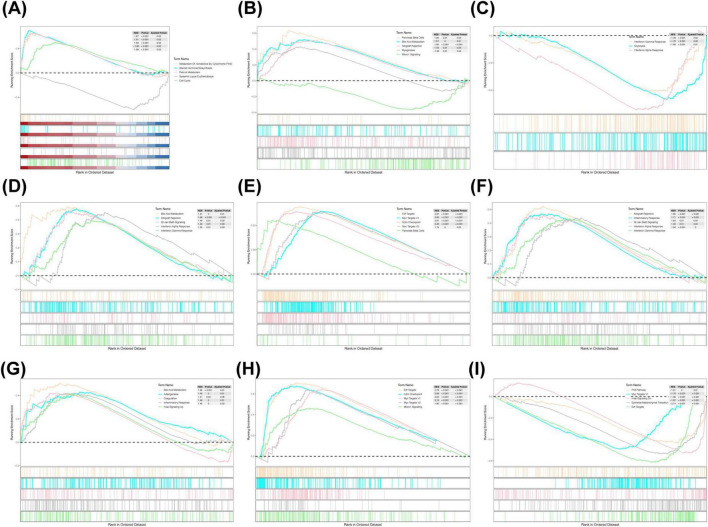
GSEA enrichment analysis. **(A)** KEGG enrichment analysis in high- and low-risk groups. **(B)** KEGG enrichment analysis of KLK8. **(C)** KEGG enrichment analysis of MFI2. **(D)** KEGG enrichment analysis of B3TNG3. **(E)** KEGG enrichment analysis of MYRF. **(F)** KEGG enrichment analysis of CREG2. **(G)** KEGG enrichment analysis of GLB1L3. **(H)** KEGG enrichment analysis of AHNAK2. **(I)** KEGG enrichment analysis of NLRP10. GSEA, Gene Set Enrichment Analysis; KEGG, Kyoto Encyclopedia of Genes and Genomes.

To explore the functional pathways associated with the prognostic genes and risk groups, GSEA was performed. The analysis revealed significant associations between the risk groups and the METABOLISM_OF_XENOBIOTICS_BY_CYTOCHROME_P450 pathway, among others (Figure 5A). For the prognostic genes, GLB1L3 and NLRP10 were enriched in the KRAS signaling pathway; MFI2, B3GNT3, and CREG2 in the Interferon Gamma Response; and KLK8, GLB1L3, and B3GNT3 in Bile Acid Metabolism (Figures 5B–I).

### 3.6 B3GNT3, GLB1L3, and CREG2, along with regulatory T cells and resting dendritic cells, may play a more significant role in NSCLC

**FIGURE 6:**
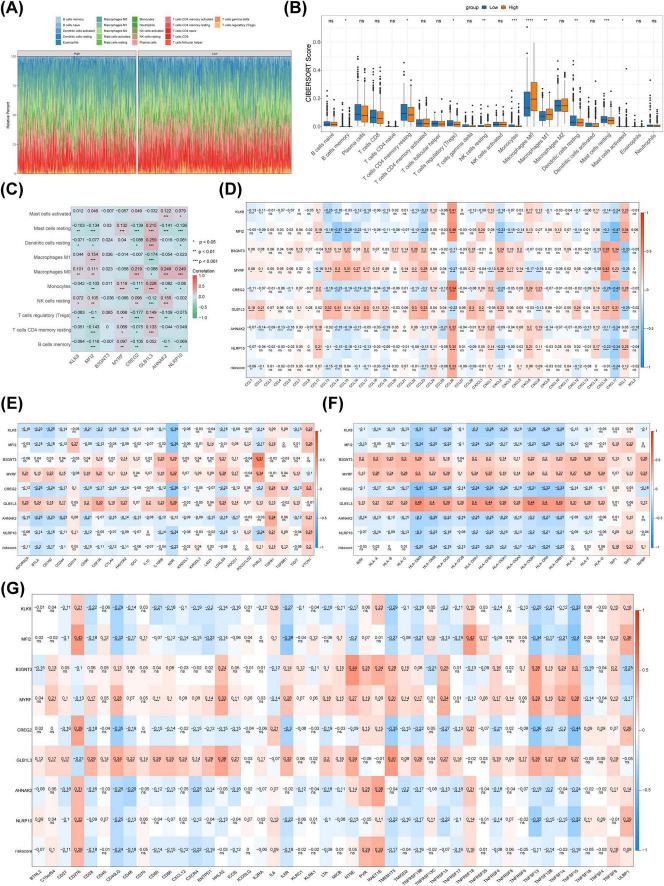
Immune infiltration analysis and response to immunotherapy. **(A)** Heatmap showing immune cell distribution in the training set. **(B)** Box plot comparing immune cell distribution between high- and low-risk groups. **(C)** Heatmap illustrating correlations between immune cell subtypes and prognostic genes. **(D)** Heatmap of correlations between chemokines and prognostic genes. **(E)** Heatmap of genetic correlations between immunostimulatory factors and prognosis. **(F)** Heatmap of histocompatibility complex and prognostic gene correlations. **(G)** Heatmap of correlations between immunosuppressants and prognostic genes.

To investigate the role of immune cells and immunotherapy in relation to prognostic genes, an immune microenvironment analysis was conducted. Immune infiltration data revealed significant disparities in the distribution of 10 immune cell types between the high- and low-risk groups (Figures 6A, B). Resting dendritic cells showed the strongest positive correlation with GLB1L3 (r = 0.259), while regulatory T cells exhibited the most negative association with CREG2 (r = -0.177) (Figure 6C). Additionally, correlations between prognostic genes and immunomolecules revealed notable relationships: CREG2 was strongly positively correlated with chemokine CCL26 (r = 0.540), PVRL2 was linked with B3GNT3 (r = 0.510), NT5E with B3GNT3 (r = 0.440), and HLA-DMA with GLB1L3 (r = 0.460) (Figures 6D–G).

**FIGURE 7:**
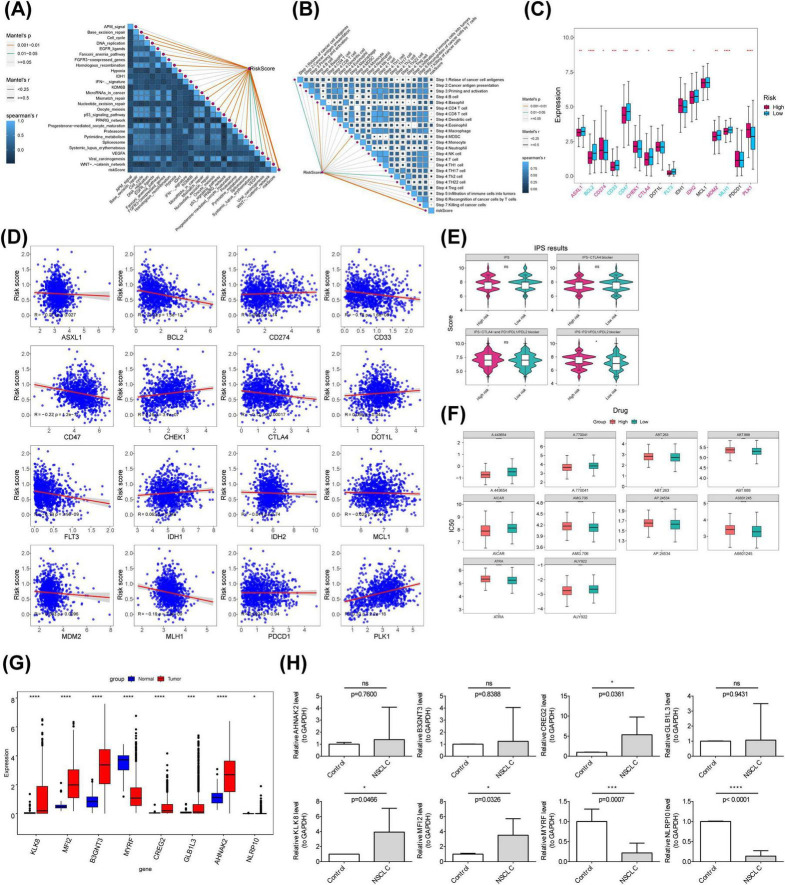
Immunotherapeutic response analysis and qRT-PCR validation. **(A)** Heatmap showing the correlation between risk scores and immune pathways in the training set. **(B)** Heatmap illustrating the correlation between risk scores and immune cycles in the training set. **(C)** Box plots of ICI-related gene expression in high- and low-risk groups. **(D)** Correlation analysis of ICI expression differential genes between high- and low-risk groups. **(E)** Comparison of IPS scores between groups. **(F)** Differential analysis of drug IC50 values between high- and low-risk groups. **(G)** Expression analysis of prognostic genes in high- and low-risk groups. **(H)** qRT-PCR validation of prognostic gene expression. ICI, immune checkpoint inhibitors. *p < 0.05; **p < 0.01; *⁣**p < 0.001; *⁣*⁣**p < 0.0001.

Further analysis identified the most prominent correlation between the risk score and immune cycle Step 4: Treg cells (r = 0.051) as well as the immunotherapy pathway Base_excision_repair (r = 0.130) (Figures 7A, B). Immune checkpoint analysis revealed significant differences in expression levels of ASXL1, BCL2, CD274, CD33, CD47, CHEK1, CTLA4, DOT1L, FLT3, MCL1, MDM2, MLH1, PDCD1, and PLK1 between the two risk groups (Figure 7C). Notably, CD274, CHEK1, DOT1L, IDH1, PDCD1, and PLK1 positively correlated with risk scores, while ASXL1, BCL2, CD33, CD47, CTLA4, FLT3, IDH2, MCL1, MDM2, and MLH1 exhibited negative correlations (Figure 7D). Moreover, IPS analysis revealed significant differences in the levels of PD1/PDL1/PDL2 blockers between the high- and low-risk groups (Figure 7E).

Chemotherapy sensitivity analysis identified 119 compounds with significantly distinct IC50 values between the two risk groups, including A.443654 and A.770041 (Figure 7F). In the TCGA-NSCLC dataset, eight prognostic genes were significantly differentially expressed between tumor and normal tissues, with MYRF showing reduced expression and the remaining genes upregulated in tumors (Figure 7G). qRT-PCR analysis confirmed the consistent expression patterns of most prognostic genes in the TCGA-NSCLC dataset, except for NLRP10. Notably, the expression levels of B3GNT3, GLB1L3, and AHNAK2 did not show significant differences, possibly due to the limited sample size (Figure 7H).

## 4 Discussion

In the era of precision medicine, the management of NSCLC has been revolutionized by targeted therapies and immunotherapies, particularly immune checkpoint inhibitors, which have significantly improved survival outcomes for certain patient subsets (35, 36). However, a substantial proportion of patients remain unresponsive to these treatments, highlighting the urgent need to identify novel therapeutic targets (3–5). Ferritinophagy, a selective autophagic process responsible for the degradation of intracellular ferritin, plays a pivotal role in ferroptosis (11, 37, 38). Targeting pathways that promote ferroptosis thus represents a promising avenue for developing innovative anticancer therapies (39, 40). In this study, we identified and validated ferritinophagy-related prognostic genes in NSCLC, constructed a robust prognostic model, and explored their functional roles and potential as predictive biomarkers for immunotherapy response. Our findings provide new insights into the interplay between ferritinophagy, ferroptosis, and the tumor immune microenvironment, offering potential strategies to enhance therapeutic efficacy in NSCLC.

Initially, NSCLC samples were scored based on FRGs from the TCGA-NSCLC dataset, revealing significant survival differences between the high- and low-scoring groups, underscoring the prognostic value of FRGs in NSCLC. While previous studies have investigated FRGs in glioma and head and neck cancers, similar research in NSCLC has been limited (41, 42). The overlapping genes from DEGs and key module genes were selected as candidate genes for further investigation. GO and KEGG enrichment analyses of these genes indicated their involvement in several key functions and pathways, including the PI3K-AKT signaling pathway, Ras signaling pathway, and cytokine-cytokine receptor interaction. The PI3K-AKT pathway, a key driver of NSCLC, not only promotes tumor growth but also suppresses ferroptosis by enhancing lipid metabolism and antioxidant defense through mechanisms such as SREBP1/SCD1-mediated lipogenesis (43). Inhibition of this pathway, as demonstrated by compounds like auriculasin, sensitizes NSCLC cells to ferroptosis, highlighting its therapeutic potential (44). Additionally, Ras signaling pathway increases reactive oxygen species (ROS) and alter lipid metabolism, promoting both cancer progression and sensitivity to ferroptosis. The enrichment of Ras signaling pathway suggests a potential crosstalk between Ras signaling and ferritinophagy, further expanding the mechanistic understanding of FRGs in NSCLC (45).

Eight prognostic genes—KLK8, MFI2, B3GNT3, MYRF, CREG2, GLB1L3, AHNAK2, and NLRP10—were identified, and a risk model based on these genes effectively predicts the survival of patients with NSCLC. Several of these genes have been shown to be significantly associated with NSCLC initiation and progression. KLK8 (human kallikrein 8) impedes lung cancer cell invasiveness by degrading fibronectin, reducing integrin signaling, and inhibiting actin polymerization, thus slowing cancer cell motility (46). Both *in vivo* studies and clinical data from patients with NSCLC reveal that elevated KLK8 levels correlate with slower tumor growth, reduced invasion, and extended time to postoperative recurrence, particularly in early-stage cases (47). KLK8’s potential role in ferritinophagy remains unexplored, but its involvement in extracellular matrix remodeling suggests it may influence iron homeostasis and tumor microenvironment dynamics (48, 49). MFI2 (melanotransferrin) accelerates NSCLC progression by promoting cell proliferation, metastasis, and invasion through miR-107-mediated NFAT5 elevation, PI3K/AKT pathway activation, and facilitating exosome-mediated progression and pre-metastatic niche formation (50). MFI2’s role in iron transport and its potential interaction with ferritinophagy warrant further investigation, as iron dysregulation is a hallmark of cancer progression. Notably, an MFI2-targeting antibody-drug conjugate is currently in phase I trials, highlighting its therapeutic potential (51). B3GNT3 is associated with poor prognosis in NSCLC, particularly in lung adenocarcinoma, where it influences cell apoptosis and holds promise as an early cancer screening marker (52, 53). Its role in cancer stem cell self-renewal and carbohydrate metabolism suggests it may intersect with ferritinophagy pathways, particularly in regulating iron-dependent cell death, which is critical for tumor suppression (54). Additionally, mutations in AHNAK2, particularly deleterious ones, are linked to improved responses to ICIs in NSCLC, opening new avenues for predictive biomarker development. Patients with NSCLC harboring AHNAK2 mutations exhibit higher tumor mutation burden (TMB), indicating enhanced tumor immunogenicity, and these mutations are associated with an activated immune microenvironment, marked by increased immune cell infiltration and activation (55). Although the roles of MYRF, CREG2, and NLRP10 in NSCLC have not been extensively studied, their involvement in other cancers has been documented. MYRF has been identified as a target of miR-199b-5p, promoting pancreatic cancer progression (56). Higher expression of CREG2 correlates with shorter survival times in esophageal squamous cell carcinoma (57). Moreover, NLRP10 is associated with a poor prognosis in endometrial cancer by inhibiting NF-κB activation and apoptosis, along with caspase-1-mediated IL-1β maturation (58). GLB1L3, expressed predominantly in the central nervous system, is involved in carbohydrate metabolism and beta-galactosidase activity, and has been implicated in schizophrenia (59, 60). No reports have yet linked GLB1L3 to malignancies. To the best of our knowledge, this study is the first to explore ferritinophagy-related genes in NSCLC. Further investigation is required to clarify the specific role of these genes in ferritinophagy within NSCLC and to assess their potential impact on iron metabolism and tumor progression.

Additionally, significant correlations between specific immune cells and prognostic genes were identified. Tumor microenvironment (TME) analysis using CIBERSORT revealed that the high-risk scoring group had lower proportions of regulatory T cells (Tregs), monocytes, resting mast cells, memory B cells, resting dendritic cells, activated mast cells, and resting memory CD4+ T cells. In contrast, this group exhibited higher proportions of M0 macrophages, M1 macrophages, and resting NK cells. These findings suggest that ferritinophagy-related genes may modulate immune cell infiltration, influencing NSCLC progression and patient outcomes. M0 macrophages, often associated with poor prognosis in cancers such as pancreatic cancer and hepatocellular carcinoma (61, 62), were enriched in the high-risk group, consistent with their pro-tumorigenic role. In contrast, M1 macrophages, which exhibit anti-tumor activity, were also elevated, suggesting a complex balance between pro- and anti-tumor immune responses. Ferritinophagy may influence macrophage polarization by regulating iron homeostasis, as iron accumulation is known to drive M2-like polarization and immunosuppression (63, 64). Targeting ferritinophagy could thus reprogram macrophages toward an anti-tumor phenotype, enhancing immunotherapy efficacy. Tregs, linked to poor prognosis in various cancers (65–67), were reduced in the high-risk group. While Tregs typically suppress anti-tumor immunity, their reduction in this context may reflect a dysregulated immune microenvironment. Ferritinophagy could modulate Treg activity by altering iron availability, which is critical for T cell function (68, 69). Further research is needed to explore whether ferritinophagy inhibition can selectively target Tregs while preserving effector T cell responses. Monocytes, associated with poor prognosis in early-stage lung squamous cell carcinoma (70), were decreased in the high-risk group, while resting dendritic cells, linked to better prognosis in lung cancer (71, 72), were also reduced. Dendritic cells play a crucial role in antigen presentation and T cell activation, and their suppression may contribute to immune evasion (73). Ferritinophagy may influence dendritic cell function by regulating iron-dependent processes such as antigen processing and cytokine production (68, 74). Resting mast cells, which regulate the TME and are associated with better prognosis in lung adenocarcinoma (75), were decreased in the high-risk group. Conversely, resting memory CD4+ T cells, linked to improved outcomes in lung adenocarcinoma (76), were also reduced. These findings suggest that ferritinophagy-related genes like GLB1L3 and AHNAK2 may modulate immune-inflammatory mechanisms in NSCLC. Elevated GLB1L3 expression was associated with increased resting dendritic cells, while high AHNAK2 expression correlated with decreased resting mast cells, highlighting their potential roles in shaping the immune landscape.

Finally, based on the GDSC database alongside established prognostic models, potential therapeutic targets and associated compounds for NSCLC were identified. These include inhibitors targeting the cell cycle (ABT.263 and AUY922), the PI3K/Akt pathway (A-443654), and the MAPK pathway (AICAR). ABT.263, a Bcl-xL/Bcl-2 inhibitor, not only induces apoptosis but may also enhance ferroptosis, by disrupting mitochondrial membrane potential and increasing lipid peroxidation (77, 78). This dual mechanism could explain its strong synergistic effect with TRAIL-inducing compounds like ONC201/TIC10, which has shown efficacy across multiple cancer types, including NSCLC (79). AUY922, an HSP90 inhibitor, demonstrates synergistic anti-cancer activity with lapatinib in HER2-positive cancers by destabilizing oncogenic client proteins and modulating stress responses, potentially including ferroptosis-related pathways (80). A-443654, an AKT pathway inhibitor, suppresses tumor growth by inducing apoptosis and may further sensitize NSCLC cells to ferroptosis through metabolic reprogramming and inhibition of survival signaling (81–83). AICAR, a purine biosynthesis intermediate, inhibits EGFR-mutant NSCLC by inducing DNA damage and apoptosis, with potential crosstalk with ferroptosis through AMPK activation and subsequent modulation of lipid metabolism (84). This approach highlights the potential of AICAR as a therapeutic agent in targeting resistant cancer cells by exploiting the interconnected pathways of apoptosis and ferroptosis (85). These findings highlight the therapeutic potential of targeting ferritinophagy-related mechanisms in NSCLC. Future research could explore combination strategies, such as pairing ferroptosis inducers with immune checkpoint inhibitors, to overcome resistance and improve outcomes in NSCLC treatment.

A key contribution of this study is the development of a novel prognostic model that effectively distinguishes survival outcomes and immunotherapeutic responses in NSCLC, integrating clinical characteristics, immune infiltration, and drug sensitivity. However, there are some limitations to this study. First, the clinical sample size was relatively small, which may affect the broad applicability of the results. Second, the molecular mechanisms regarding the FRGs involved in NSCLC are still incompletely understood, and thus further in-depth studies on the significance of these genes in diagnosis and treatment are needed. In the future, we plan to collect more clinical samples and conduct large-scale, prospective randomized controlled trials to further validate the results of this study and optimize treatment strategies.

## 5 Conclusion

In this study, we developed a novel prognostic model using machine-learning techniques and TCGA data to predict overall survival in NSCLC patients. This model not only accurately estimates survival probabilities but also identifies a risk score strongly associated with the immune microenvironment and clinicopathological features. Importantly, our findings highlight the critical role of ferritinophagy-related genes in NSCLC prognosis and their potential influence on ferroptosis and immune regulation. Based on these findings, prognostic genes may serve as potential therapeutic targets to drive the development of novel therapeutic agents. In addition, the application of this model not only provides new ideas for the early diagnosis of NSCLC, but also provides an important basis for the development of personalized therapeutic regimens, which is of great clinical significance.

## Data Availability

The datasets analyzed for this study can be found in the UCSC Xena website, TCGA-NSCLC (https://xena.ucsc.edu/), Gene Expression Omnibus (GEO) (https://www.ncbi.nlm.nih.gov/geo/), GeneCards database (https://www.genecards.org/).

## Footnotes

1
https://xena.ucsc.edu/

2
https://www.ncbi.nlm.nih.gov/geo/

3
https://www.genecards.org/

4
https://string-db.org/

5
https://www.gsea-msigdb.org/gsea/msigdb

6
http://cis.hku.hk/TISIDB/

7
https://tcia.at/home
